# Multi-Omics Revealed the Effects of Different Feeding Systems on Rumen Microorganisms, Cellulose Degradation, and Metabolites in Mongolian Cattle

**DOI:** 10.3390/ani15121774

**Published:** 2025-06-16

**Authors:** Kexin Jiang, Jianfei Ma, Junzhao Xu, Ying Zhang, Huaxin Niu

**Affiliations:** College of Animal Science and Technology, Inner Mongolia Minzu University, Tongliao 028000, China; 13634709903@163.com (K.J.); 18648043034@163.com (J.M.); xujunzhao1124@163.com (J.X.); 15947157659@163.com (Y.Z.)

**Keywords:** cellulose degradation, Mongolian cattle, microbiome, metabolomic, rumen

## Abstract

Rumen microbiota is crucial for cellulose degradation and nutrient metabolism in ruminants. Different feeding systems like grazing and housed feeding can significantly impact it. Mongolian cattle show unique cellulose degradation ability, but functional changes under different feeding conditions are unclear. This study aims to investigate the effects of grazing and housed feeding on rumen microbiota, cellulose degradation, and metabolism in Mongolian cattle. In a 90-day trial, 12 female Mongolian cattle were divided into grazing (F group) and housed feeding (S group). Rumen samples were collected to analyze fermentation parameters, enzyme activities, microbiomes, and metabolomes. The F group had higher acetate, cellulase, xylanase, and β-glucosidase activities (*p* < 0.05). Bacteroidota and *Prevotella* were more abundant (*p* < 0.05), while Firmicutes and *Ruminococcus* were less abundant (*p* < 0.05) in the F group. Carbohydrate metabolic pathways and CAZymes (GH2, GH10) were upregulated in the F group, while the S group had enriched purine metabolic pathways and CAZyme (GH31). A total of 64 differential metabolites were found, with subaphylline upregulated in the F group and L-arogenate in the S group (*p* < 0.05). Grazing increased cellulose degradation and subaphylline production in Mongolian cattle, while housed feeding improved starch utilization efficiency and fat synthesis. These findings provide a basis for optimizing feeding strategies and improving fibrous feed resource utilization in Mongolian cattle.

## Simple Summary

This study investigates how different feeding methods (grazing vs. housed feeding) affect the rumen microbial community, cellulose degradation, and metabolic profiles in Mongolian cattle. The results show that grazing enhances fiber degradation and specific metabolite production, while housed feeding improves starch utilization efficiency and fat synthesis. These findings provide insights for optimizing feeding strategies and improving fiber feed resource utilization in Mongolian cattle.

## 1. Introduction

Mongolian cattle are ancient breeds with a long history of tolerance to cold and roughage, resistance to disease, and adaptation to harsh environments [1]. Native to the Mongolian Plateau, Mongolian cattle were once prevalent across the Inner Mongolia Autonomous Region and the northeast, northwestern, and southern provinces of China. The most famous breed is found in Ujimqin, Xilingol League, in Inner Mongolia. The Inner Mongolia Autonomous Region is an important production area for Mongolian cattle, which inhabit vast grasslands. Mongolian cattle have traditional husbandry practices. Traditionally, they are mainly raised through year-round grazing, relying on natural grassland resources [2]. To address over-grazing and promote sustainable grassland management, recent years have seen an increasing number of Mongolian cattle being raised through total mixed ration housed feeding systems. Although the housed feeding method makes full use of resources like corn stalks, silage, and other crop residues and relieves pasture pressure to a certain extent, grazing is still the main feeding method for Mongolian cattle. It enables the cattle to feed on a variety of pasture grasses in their natural environment, making full use of plant fibers and promoting the ecological and forage sustainability of the balance between grass and livestock.

The rumen is a rich and diverse microbial ecosystem that primarily consists of anaerobic bacteria, protozoa, fungi, archaea, and phages [3]. Rumen microbes are integral to fiber feed breakdown and nutrient transformation, and they also play an extremely important role in the digestive process of ruminants [4]. These microbiota break down cellulose via fermentation into volatile fatty acids (VFAs), such as acetate, propionate, and butyrate, which are the main sources of energy for ruminants [5]. Additionally, the ability of the rumen to digest fiber-rich feeds and convert them into various metabolites is an important aspect. These metabolites are utilized by microbiota. Meanwhile, they are also absorbed by ruminants for the maintenance of their growth. Specifically, these processes represent the connection among the host, rumen microbiota, and ration levels. Many researchers have focused on the fact that rumen microbes and metabolites in ruminants are affected by changes in the type of forage and the environment. He et al. [6] demonstrated that compared with yaks fed full corn silage and supplemented with 3 kg concentrate per head per day, the natural grazing group of yaks feeding on natural forage had higher rumen microbial diversity. Fu et al. [7] demonstrated that compared with Tan sheep fed a concentrate-supplemented diet, the grazing group of Tan sheep consuming natural forage had higher rumen cellulolytic bacteria. It was also shown [8] that the rumen fermentation parameters, microbial composition, and metabolome of yaks fed diets with concentrate-to-forage ratios of 50:50, 65:35, and 80:20 have all undergone significant changes. Additionally, Liu et al. [9] revealed that yak rumen bacterial communities and metabolomes exhibit distinct responses to feed type shifts, with forage-based diets enhancing the abundance of fiber-degrading genera and modulating metabolic pathways related to protein digestion, purine metabolism, and fatty acid biosynthesis.

However, there is still a scarcity of reports on the rumen microbiota and its cellulose degradation function in Mongolian cattle grazing during winter. Most previous studies [2,10] on Mongolian cattle have focused on other aspects, such as rumen functional development and microbial succession rather than specifically addressing the rumen microbiota and cellulose degradation function in winter-grazing Mongolian cattle. Therefore, in this study, metagenomics and Liquid Chromatography–Mass Spectrometry (LC-MS) nontargeted metabolomic techniques were used to assess rumen microbial communities, digestive characteristics, and metabolites of Mongolian cattle under grazing natural hay and housed feeding Total Mixed Ration (TMR). It was hypothesized that the two feeding systems, grazing and housed feeding, would regulate the structure of rumen microbial communities and the expression of functional genes, thereby influencing the degradation efficiency of fibrous feed and the composition of metabolites in Mongolian cattle, and ultimately leading to differences in rumen fermentation characteristics and energy utilization directions.

## 2. Materials and Methods

### 2.1. Animal and Experiment Design

In total, 12 healthy local Mongolian female cattle (3–4 years old; weighed 460 ± 35 kg) were selected as test animals in Uragai Ranch, Xilingol League, Inner Mongolia Autonomous Region. These cattle were divided into a housed feeding group (S group) and a grazing group (F group). Each group contained six animals, and a completely randomized experimental design was adopted. The F group of Mongolian cattle grazed in the Uragui grasslands and fed on natural pasture (the main types of pasture are *Leymus chinensis*, *Filipendula ulmaria*, *Festuca capillata*, etc.). Mongolian cattle in the S group were kept in semi-open pens and fed a basal ration. The basal ration was formulated according to NRC guidelines [11] (Table S1). The experiment was conducted in winter, with a 10-day pretrial period followed by a 90-day formal experimental period. The Mongolia cattle in the S group were fed with 12 kg of TMR every day. All animals were free to feed and drink.

At the conclusion of this experiment, rumen fluid was collected via a flexible oral gastric tube equipped with a metal filter prior to the morning feeding. A vacuum rumen tube (Wuhan Colibri Ranching Technology Co., Ltd., Wuhan, China) was used to collect rumen fluid through the mouth, and 200 mL of rumen fluid was first extracted and discarded. Then, 200 mL of rumen fluid was extracted, filtered through four layers of sterilized gauze, and aliquoted into 15 mL cryopreservation tubes, which were stored in a liquid nitrogen tank and then sent to Meiji Biologicals, Inc. (Tokyo, Japan), within 2–4 h with dry ice for microbial DNA extraction and sequencing analysis.

### 2.2. Determination of Feed Nutrient Content

The levels of crude protein (CP; method 2001.11), neutral detergent fiber (NDF; method 2002.04), acid detergent fiber (ADF; method 973.18) and starch (starch; method 996.11) were measured following the procedures of AOAC (2005) [12]. Calcium and phosphorus (P) in feed samples were analyzed using inductively coupled plasma mass spectroscopy (AOAC, 2005; method 985.01) [12].

### 2.3. VFA and Cellulose Degrading Enzyme Activity Index Measurements

pH: The pH of the rumen fluid was determined using a PHSJ-3F laboratory pH meter (Yantai Stark Instrument Co., Ltd., Yantai, China). Before measurement, the pH meter was calibrated with standard buffer solution. After taking the rumen fluid out of storage and allowing it to thaw completely, the electrode was immersed in the thawed rumen fluid, and the pH was recorded after the reading stabilized.

VFAs: The concentration of VFAs was determined using an Agilent 1160 gas chromatograph following the method described by Erwin et al. [13] (Agilent Technologies, Santa Clara, CA, USA).

Cellulose-degrading enzyme activity: Following the method described by Guo et al. [14], a kit from Jiangsu Enzyme Immunity Industry Co., Ltd. (Nanjing, China) was used. Ruminal digestive enzymes (cellulase, xylanase, and β-glucosidase) were measured via ELISA.

### 2.4. DNA Extraction and Macrogenomic Determination

The rumen fluid obtained above was used for microbial DNA extraction using an MP kit (MP Biomedicals, Santa Ana, CA, USA). DNA concentration and purity were measured using a Nanodrop spectrophotometer (Thermo Fisher Scientific, Waltham, MA, USA). The extracted DNA then underwent quality testing, fragmentation, and end repair to construct a sequencing library. The constructed library was subjected to quality and concentration control using agarose gel electrophoresis and qPCR, mixed with DNA polymerase and fluorescently labeled primers, and sequenced for DNA fragments on the Nova Seq platform; the sequencing was performed by Meiji Bio (NovaSeq platform, Shanghai, China).

### 2.5. Macrogenome Sequencing and Analysis of Rumen Microorganisms

The fragments from sequencing were filtered using the Fastp software (v 0.20.0) to obtain high-quality clean reads for subsequent analysis. The sequences were spliced and assembled using the software MEGAHIT (v 1.1.2) [15], followed by open read frame (ORF) prediction of the contigs using Prodigal (v 2.6.3). Genes with nucleic acid lengths of 100 bp or more were selected and converted into amino acid sequences. Nonredundant gene collections were created using the CD-HIT (v 4.6.1, default parameters: 90% identity, 90% coverage) [16] software to obtain the base sequences of the genes in the nonredundant gene sets. Next, SOAPaligner (v 2.21) was used to compare high-quality reads with nonredundant gene sets for each sample to collect data on gene prevalence across samples. All online databases and software were accessed in August 2023. Information on species composition was obtained using the software BLASTP (v 2.2.28+, http://blast.ncbi.nlm.nih.gov/Blast.cgi, accessed on 2 August 2023) compared to the NR database (https://ftp.ncbi.nlm.nih.gov/blast/db/FASTA/, accessed on 4 August 2023). The Kyoto Encyclopedia of Genes and Genomes (KEGG) database (https://www.genome.jp/kegg, accessed on 8 August 2023) was compared using the software BLASTP (v 2.2.28+, http://blast.ncbi.nlm.nih.gov/Blast.cgi, accessed on 10 August 2023) to obtain the corresponding KEGG annotation profiles of the genes and perform statistical analysis. An annotation profile of carbohydrate-active enzyme-encoding genes was obtained using hmmscan against the CAZy database (http://www.cazy.org/, accessed on 14 August 2023).

### 2.6. Non-Targeted Metabolome Sequencing

A sample of rumen fluid was collected in a centrifuge tube, injected with a water-acetonitrile mixture, vortexed and mixed for 30 s, and left undisturbed in an ice bath before centrifugation was performed. Simultaneously, an equal volume of the sample was mixed to make a quality control (QC) sample. The samples were separated using a Hypesil Gold column (manufactured by Thermo Fisher Scientific, Waltham, MA, USA) in positive and negative ion modes and screened for particles with mass-charge ratios between 70 and 1000 using a Q ExactiveTM HF-X mass spectrometer (manufactured by Thermo Fisher Scientific, Waltham, USA) in positive and negative ion scanning modes. After finalizing the onboarding process, the unprocessed data were analyzed using the Progenesis QI software (v 2.0, Waters Corporation, Milford, MA, USA). In this step, variables from the quality control samples that had a relative standard deviation (RSD) greater than 30% were filtered out, resulting in refined data matrix postprocessing. The mass spectral information was matched with HMDB and Metlin as well as Meggie to construct libraries. Information on metabolites was obtained. The processed matrices were submitted to the Megibio cloud platform (https://cloud.majorbio.com, accessed on 4 August 2023) for evaluation, and the data were processed and analyzed using the partial least squares discriminant analysis (PLS-DA) software, metaX (v 1.4.2), to obtain the variable importance projection (VIP), retention time (RT), and mass-to-charge ration (m/z) of the metabolites. Statistical analysis of metabolites between the two groups was conducted to calculate the multiplicity of differences in metabolites between different groups [17]. VIP > 1.0, *p* < 0.05, FC > 1.5, or FC < 0.667 were used as the screening criteria to identify the differential metabolites, and the pathway information on metabolite involvement was obtained according to the KEGG database (https://www.kegg.jp/, accessed on 16 August 2023) [18]. Pathway enrichment was conducted using the Python software package (v 3.0) and the key biological pathways related to the experimental treatments were identified by conducting Fisher’s exact test.

### 2.7. Statistical Analysis

Prior to statistical analysis, the normality of rumen fermentation parameters, cellulase activity, and α diversity index was assessed using the Shapiro–Wilk test. Homogeneity of variances was evaluated using Levene’s test. All parameters satisfied the assumptions of normality (Shapiro–Wilk *p* > 0.05) and homogeneity of variances (Levene’s *p* > 0.05). Statistical differences were analyzed using an independent sample *t*-test via SPSS 27.0 (IBM, Armonk, NY, USA). Significance was set at *p* < 0.05, and results were presented as the mean ± standard error of the mean (SEM).

The rumen microbial phyla and genera were compared using the Welch *t*-test, with the FDR adjusted *p* < 0.05 being considered as significantly different. The KEGG pathways and CAZymes were compared by the Wilcoxon rank-sum test, with the FDR adjusted *p* < 0.05 being considered significantly different. Using PLS-DA and Student’s *t*-test, the significance of metabolite difference in each group was analyzed, and the difference was statistically significant with *p* < 0.05. Pathway enrichment was conducted using the Python software package and the difference was statistically significant with *p* < 0.05. The correlations between different metabolites and bacterial communities were calculated by Pearson correlation analysis using the R program (v 3.6.1), and *p* < 0.05 indicated significant differences.

## 3. Results

### 3.1. Rumen Fermentation Parameters and Cellulose-Degrading Enzymes

The pH of the rumen fluid was lower, but the total VFA concentration was higher (*p* > 0.05) in the F group than in those in the S group (Table 1), whereas the concentration of acetate in the rumen fluid of the F group was significantly greater (*p* < 0.05). The activities of rumen fluid cellulase, xylanase, and β-glucosidase (β-GC) in the F group were significantly greater than those in the S group (*p* < 0.05).

### 3.2. Metagenome Sequencing Information

Metagenome sequencing was performed on 12 Mongolian cattle rumen fluid samples (Table S2), and 1,051,056,016 sequences were obtained after disembarking the machine, with an average of 87,588,001 sequences per sample. After decontamination, 1,030,399,840 high-quality sequences were obtained, with an average of 85,866,653 sequences per sample, and the data were spliced and assembled to yield 16,951,894 contigs, with an average of 1,412,657 per sample, of which the N50 was 631 on average.

### 3.3. Rumen Microbial Diversity

In this experiment (Table 2), 12 samples from the F group and the S group were subjected to metagenome sequencing, and α diversity analysis revealed that the Abundance-based Coverage estimator (ACE) index and Simpson index of the F group were greater than that of the S group, but the difference was not significant (*p* > 0.05).

### 3.4. Rumen Microbial Composition

**Figure 1:**
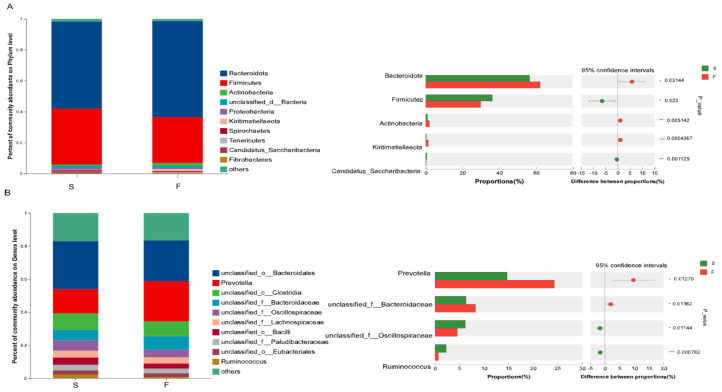
Ruminal microbial bacterial flora composition of Mongolian cattle with different feeding systems and significant differences at the phylum (**A**) and genus level (**B**). F, grazing; S, housed feeding. * 0.01 < *p* ≤ 0.05, ** 0.001< *p* ≤ 0.01, *** *p* ≤ 0.001.

Bacteria in the rumen of Mongolian cattle were analyzed, and 156 phyla, 261 classes, 461 orders, 926 families, 3388 genera, and 16,927 species were identified. At the phylum level (Figure 1A), the top 10 species in terms of abundance were selected. The dominant phyla in all samples were Bacteroides (mean abundance of 62.10% in the F group, mean abundance of 56.32% in the S group) and Firmicutes (mean abundance of 29.71% in the F group, mean abundance of 36.13% in the S group). In comparison with the S group, the F group showed higher (*p* < 0.05) relative abundances of Bacteroidota, Actinobacteria, and Kiritimatiellaeota and lower (*p* < 0.05) relative abundances of Firmicutes and Candidatus_Saccharibacteria.

The top 10 genera were analyzed at the genus level (Figure 1B). *Unclassified_o__Bacteroidales* (mean abundance 28.97% in the F group, mean abundance 24.71% in the S group), *Prevotella* (mean abundance 24.34% in the F group, mean abundance 14.72% in the S group), *unclassified__c__Clostridia* (mean abundance 8.89% in the F group and 10.12% in the S group), and *unclassified__f__Bacteroidacea* (mean abundance 8.26% in the F group and 6.33% in the S group) were the dominant genera in all samples. In comparison with the S group, the F group showed higher (*p* < 0.05) relative abundances of *Prevotella* and *unclassified_f__Bacteroidaceae* and lower (*p* < 0.05) relative abundances of *unclassified_f__Oscillospiraceae* and *Ruminococcus*.

### 3.5. Rumen Microbial KEGG Pathway

**Figure 2:**
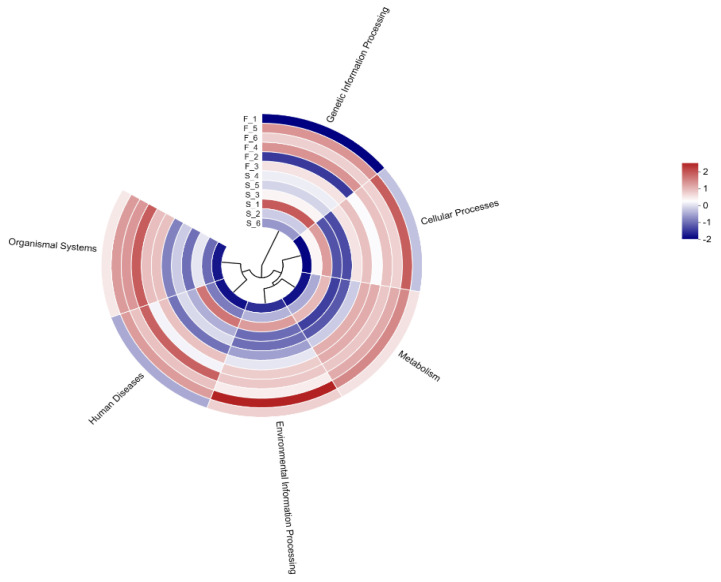
Functional abundance circos plot at the level 1 hierarchy level based on the KEGG database. F, grazing; S, housed feeding.

We performed KEGG functional annotation of rumen microbial functions in Mongolian cattle using different feeding systems. Among the KEGG level 1 pathways (Figure 2), metabolism (49.96%) had the highest mean relative abundance in both groups, followed by genetic information processing (19.66%), environmental information processing (11.64%), cellular processes (8.58%), human diseases (6.21%), and organismal systems (3.89%).

**Figure 3:**
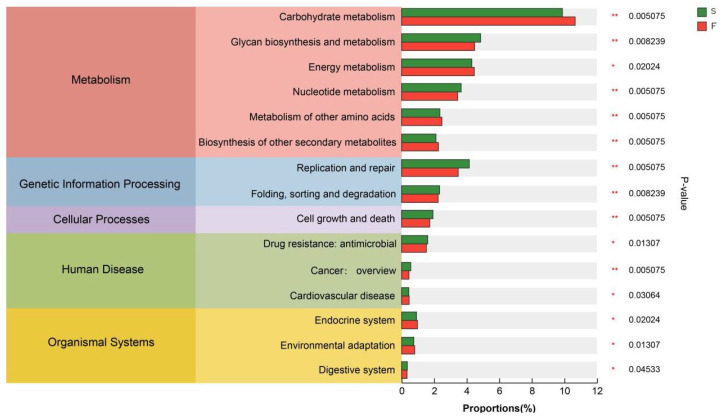
Comparison plot of functional abundance at the level 2 hierarchy level based on the KEGG database. F, grazing; S, housed feeding. * 0.01 < *p* ≤ 0.05, ** 0.001< *p* ≤ 0.01.

Among the secondary pathways (Figure 3), a comparison of the functional differences in the KEGG pathways between the two feeding systems revealed that carbohydrate metabolism, energy metabolism, and biosynthesis of other secondary metabolites were significantly greater in the F group than in the S group (*p* < 0.05), and that glycan biosynthesis and metabolism, nucleotide metabolism, replication and repair, and the growth and death of cells were significantly greater in the S group than in the F group (*p* < 0.05).

**Figure 4:**
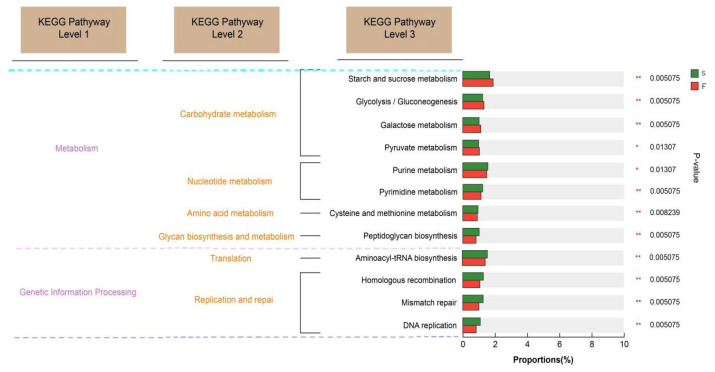
Comparison plot of functional abundance at the 3rd level of hierarchy based on the KEGG database. F, grazing; S, housed feeding. * 0.01 < *p* ≤ 0.05, ** 0.001 < *p* ≤ 0.01. Purple font represents KEGG Pathway level 1, orange font represents KEGG Pathway level 2, and dashed lines in different colors are used to divide KEGG Pathway level 1.

In the tertiary pathway comparison (Figure 4), glycolysis, galactose metabolism, and pyruvate metabolism were significantly greater (*p* < 0.05) in the F group than in the S group, and purine metabolism, pyrimidine metabolism, and peptidoglycan biosynthesis were significantly greater (*p* < 0.05) in the S group than in the F group.

### 3.6. Rumen Microbial CAZymes

**Figure 5:**
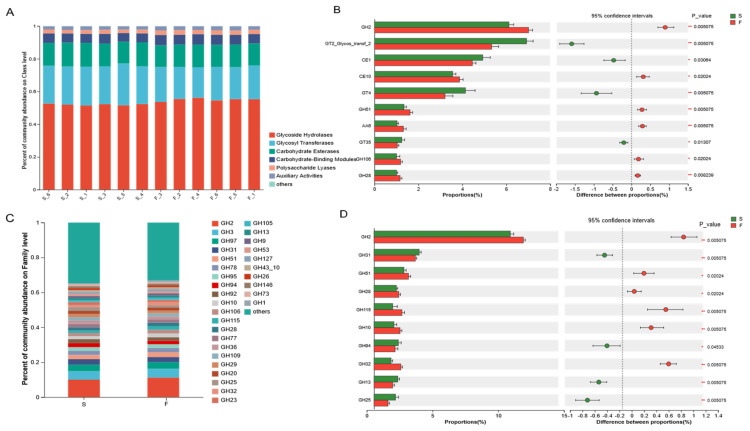
Abundance of rumen metagenome CAZymes in Mongolian cattle under different feeding systems and comparison between the two groups (family level). (**A**) Histogram of CAZymes abundance. (**B**) Significant differences in CAZymes. (**C**) Histogram of GH family abundance. (**D**) Significant differences in the GH family. F, grazing; S, housed feeding. * 0.01 < *p* ≤ 0.05, ** 0.001 < *p* ≤ 0.01.

A comparison of the genes with the carbohydrate-active enzyme database, CAZy, and carbohydrate-active enzyme annotation results revealed that at the class level (Figure 5A), the highest relative abundance of GHs (53.64%) was found in F and S groups, followed by GTs (21.85%), CEs (13.90%), CBMs (5.87%), PLs (2.44%), and AAs (2.26%).

The composition of the GH family (abundance > 0.01%) was as follows (Figure 5C): GH2 (10.01% in the S group, 11.20% in the F group), GH3 (4.92% in the S group, 5.03% in the F group), GH97 (3.66% in the S group, 3.77% in the F group), GH31 (3.14% in the S group, 2.94% in the F group), GH51 (2.37% in the S group, 2.69% in the F group), GH78 (2.39% in the S group, 2.46% in the F group), and GH95 (2.13% in the S group, 2.17% in the F group).

Differences in the genes encoded by CAZymes were compared between the two groups (Figure 5B). The results revealed that the relative abundances of GH2, CE10, GH51, and AA6 were significantly higher (*p* < 0.05) in the F group than in the S group and that the relative abundances of GT2, CE1, GT4, and GT35 were significantly higher (*p* < 0.05) in the S group than in the F group. The most dominant Cazy in the rumen microbiota of Mongolian cattle for degrading cellulose is the GH Family.

A comparison between the two groups of the GH family was performed (Figure 5D), and the results revealed that the relative abundances of GH2, GH51, GH28, GH115, and GH10 in the F group were significantly greater than in the S group (*p* < 0.05) and that the relative abundance of GH9 in the F group was higher than that in the S group; however, the difference was not significant (*p* > 0.05). The relative abundances of GH31, GH94, GH13, and GH25 in the S group were significantly higher (*p* < 0.05) than those in the F group.

### 3.7. Rumen Fluid Metabolic Profiles

**Figure 6:**
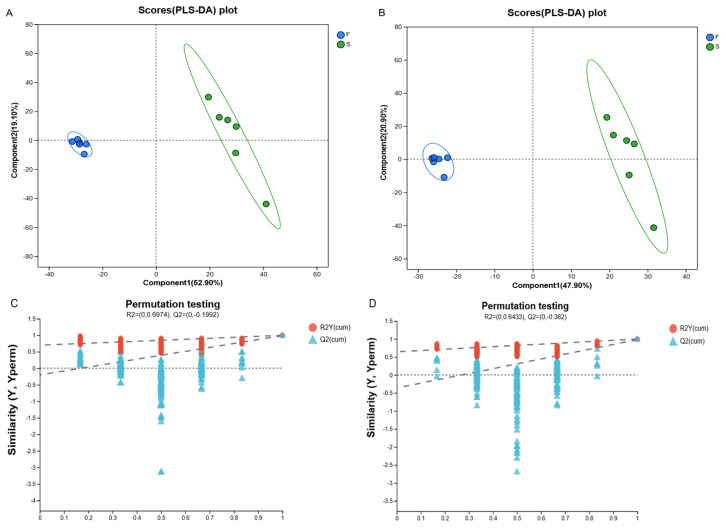
PLS-DA analysis and replacement tests of anions and cations in samples in different groups. The abscissa represents the permutation retention of the permutation test. The ordinate represents the value of the R2 (red) and Q2 (blue) permutation tests. The two dashed lines represent the regression lines of R2 and Q2. (**A**) PLS-DA scores of Mongolian cattle metabolites in the cationic mode. (**B**) PLS-DA score plot of metabolites from Mongolian cattle in anionic mode. (**C**) PLS-DA alignment test of metabolites. (**D**) PLS-DA alignment test. F, grazing; S, housed feeding.

To compare the differential metabolites of Mongolian cattle rumen fluid under different feeding systems more precisely (Figure 6A,B), the classification effect of the two feeding systems was demonstrated using PLS-DA score plots; the two groups showed a bilevel distribution, which indicated that the classification effect was significant, and the samples were clustered, implying that a significant differentiation occurred in the rumen microbiota of the Mongolian cattle in the F and the S groups. The PLS-DA replacement test showed that all R2Y values were close to 1, and R2Y was >Q2 (Figure 6C,D). The Q2 regression straight line intercept with the Y-axis was less than 0, indicating that the obtained PLS-DA model was stable and did not have a fitting phenomenon. Additionally, the samples shown in the PLS-DA score plot were all located within the sample confidence circles, demonstrating that the PLS-DA validation model had a high level of confidence. The results were reasonable and reliable and thus were used for subsequent analysis.

**Figure 7:**
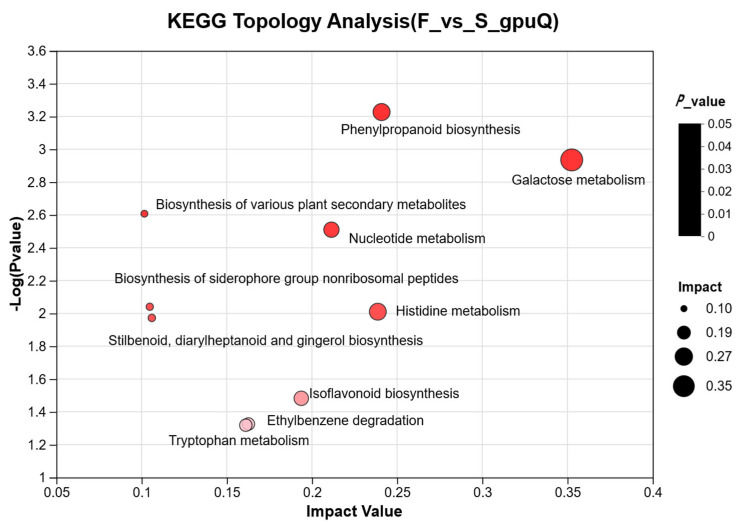
Metabolic pathway enrichment analysis. Note: Each bubble in the figure represents a KEGG pathway; the horizontal axis represents the size of the relative importance of the metabolite in the pathway in terms of the impact value; the vertical axis represents the enriched significance of the metabolite involved in the pathway −log10 (*p*-value); the size of the bubble represents the value of the impact value; the larger the bubble is, the greater the importance of the pathway. F, grazing; S, housed feeding.

The criteria to screen the differential metabolites in the F and the S groups were as follows: VIP > 1, P < 0.05, FC > 1.5, or FC < 0.667; based on the criteria, 64 differential metabolites were screened in positive and negative ion modes (Table S3), and based on the categorization information, the differential metabolites were classified into organic heterocyclic compounds, lipids and lipid-like molecules, organic acids and derivatives, nucleosides, nucleotides and analogs, alkaloids and derivatives, etc. In total, 25 metabolites, including Daucol, Verbenalol, and 4-Oxoretinol, were significantly upregulated, and 39 metabolites, including Prodolic acid and N1-Acetylspermine, were significantly downregulated in the rumens of the Mongolian cattle in the F group compared to those in the S group. Analysis of metabolic pathways (Figure 7) revealed the top 10 significantly affected pathways (*p* < 0.05) related to galactose, histidine, nucleotide, tryptophan, β-alanine metabolism, biologs of various plant secondary metabolites, phenylalanine biologs, phenylalanine, tyrosine and tryptophan biologs, and isoflavone biosynthesis in the F and the S groups, respectively.

### 3.8. Correlation Analysis of Metabolites with Rumen Bacteria

**Figure 8:**
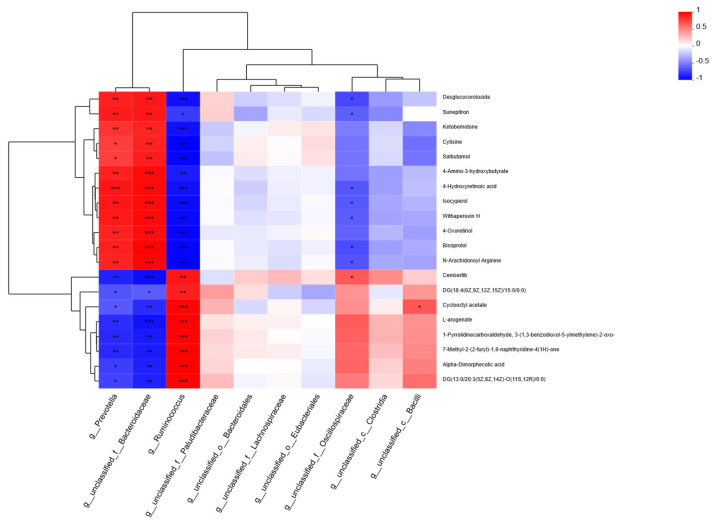
Heatmap of the correlations between dominant rumen microflora genera and differential metabolites in Mongolian cattle subjected to different feeding systems. * 0.01< *p* ≤ 0.05, ** 0.001< *p* ≤ 0.01, *** *p* ≤ 0.001.

A correlation heatmap was created using Pearson’s correlation coefficient to examine the relationships between prominent microbial genera and various metabolites (Figure 8). A strong correlation was found between the dominant microbial genera and the differential metabolites. The taxon *g_Prevotella* showed a significant positive correlation with cytisine and salbutamol, and a significant positive correlation with desglucocoroloside, sunepitron, ketobemidone, 4-amino-3-hydroxybutyrate, 4-hydroxyretinoic acid, isocyperol, withaperuvin H, 4-oxoretinol, bisoprolol, and N-arachidonoyl arginine. Additionally, DG (18:4(6Z,9Z,12Z,15Z)/15:0/0:0), cyclooctyl acetate, alpha-dimorphecolic acid, and DG(13:0/20:3(5Z,8Z,14Z)-O(11S,12R)/0:0) showed a significant negative correlation with *g_Prevotella*. Cenisertib, L-arogenate, 1-pyrrolidine carboxaldehyde, and 7-methyl-2-(2-furyl)-1,8-naphthyridine-4(1H)-one showed a highly significant negative correlation with *g_Prevotella*. The taxon *g_unclassified_f__Bacteroidaceae* showed highly significant features with desglucocoroloside, sunepitron, ketobemidone, cytisine, salbutamol, 4-amino-3-hydroxybutyrate, 4-hydroxyretinoic acid, isocyperol, withaperuvin H, 4-oxoretinol, bisoprolol, and N-arachidonoyl arginine. A significant negative correlation was found between *g_unclassified_f__Bacteroidaceae* and DG (18:4(6Z,9Z,12Z,15Z)/15:0/0:0), and a highly significant negative correlation was found between *g_unclassified_f__Bacteroidaceae* and cenisertib, cycloctyl acetate, L-arogenate, 7-methyl-2-(2-furyl)-1,8-naphthyridine-4(1H)-one, alpha-dimorphecolic acid, and DG(13:0/20:3(5Z,8Z,14Z)-O(11S,12R)/0:0). The taxon *g_Ruminococcus* showed a highly significant positive correlation with cenisertib, DG(18:4(6Z,9Z,12Z,15Z)/15:0/0:0), cyclooctyl acetate, L-arogenate, alpha-dimorphecolic acid, and DG(13:0/20:3(5Z,8Z,14Z)-O(11S,12R)/0:0); a significant negative correlation with sunepitron; and a highly significant negative correlation with desglucocoroloside, ketobemidone, cytisine, salbutamol, 4-amino-3-hydroxybutyrate, 4-hydroxyretinoic acid, isocyperol, withaperuvin H, 4-oxoretinol, bisoprolol, and N-arachidonoyl arginine.

## 4. Discussion

Ruminal pH is an important indicator used to evaluate the health of the rumen and visualize the internal setting of the rumen, which is affected by the combination of feed type, ration intake, saliva secretion, and the accumulation of organic acids [19]. VFA produced by microbiota in the rumen through fermentation is a key form of energy for ruminants, as they provide 70% of metabolic energy [5]. Microbiota initially degrade crude fiber, starch, and soluble sugars into pyruvic acid, which is then converted into various VFAs (acetate, propionate, butyrate, isobutyrate, valerate, and isovalerate) through distinct metabolic pathways [20]. Previous research [21,22] have indicated that an increased presence of cellulose, hemicellulose, and lignin in feedstuffs enhances the production of acetate during rumen fermentation. Furthermore, as acetate constitutes a major portion of TVFA, its rise contributes to an overall increase in TVFA levels. In the present study, the KEGG pathway analysis revealed that carbohydrate and pyruvate metabolism were significantly upregulated in the F group, indicating that a greater amount of crude fiber serves as a substrate for microbial fermentation and VFA synthesis in Mongolian cattle under grazing conditions. Consequently, in comparison to the S group, the F group exhibited higher TVFA levels and a marked increase in acetate concentration. Although the two feeding systems, i.e., grazing and housing, affected the rumen pH, currently, we could only observe that the rumen pH was maintained within a certain range, but a more precise definition of this range and the measurement of the buffer capacity of the rumen microbiota will be the focus of our follow-up research to better understand its adaptability to the different feeding modes and the stability of the internal environment of the rumen.

The rumen ecosystem is focused primarily on the breakdown of plant fibers through a diverse community of microbiota. This function allows ruminants to digest feed, thus emphasizing its importance for human needs, as it is the main contributor to the conversion of plant biomass into essential products (milk, meat, and fiber products) [23]. Plant cell walls are composed of biopolymers and structural proteins such as cellulose, hemicellulose, lignin, and pectin, with cellulose being the main component in plant cell walls [24]. Cellulases produced by rumen fibrolytic bacteria can cleave the β-1,4 bonds in the cellulose chain, thus degrading cellulose polymers [25]. β-Glucosidase can effectively degrade cellobiose and cellotriose [26] and convert cellulose into available glucose. Xylanases are an important class of hemicellulases that play a key role in breaking down the complex structure of xylan [27]. These abundant CAZymes facilitate the efficient degradation of cellulose. It has been reported [28] that the supplementation of isovalerate enhances cellulase activity and leads to an increase in total rumen VFA concentration in calves. In this study, isovalerate, cellulase, xylanase, and β-glucosidase (β-GC) levels were significantly higher in the F group compared to those in the S group, indicating that Mongolian cattle can stimulate cellulase activity, accelerate the fixation of cellulase, and enhance the ability to digest cellulose and hemicellulose under grazing conditions.

The sequencing results revealed that different feeding systems affected the composition of the rumen bacterial community. Ruminal microbiota play an extremely important role in ruminants by breaking down cellulose and hemicellulose into simple sugars and short-chain fatty acids through fermentation. Rumen bacteria play a key role in the degradation of cellulose and hemicellulose [25]. Many studies in ruminants [9,29], have shown that the dominant phyla of rumen microbiota are Bacteroidota and Firmicutes, regardless of their ration composition. The results of this experiment revealed that Bacteroidota and Firmicutes were the dominant phyla in the rumens of Mongolian cattle in all the samples from the different feeding systems. Some studies [30] have found that Bacteroidota can degrade crude cellulose, but Bacteroidota may be affected by carbohydrate structure. Firmicutes are the main degraders of complex carbohydrates [31]. Studies [9,32], have also found that Firmicutes significantly increased and Bacteroidota significantly decreased in the rumen microbiota of ruminants as the level of concentrate in the feed increased. The results of this study revealed that the abundance of Bacteroidota significantly increased in the rumen of the Mongolian cattle in the F group (higher crude fiber content in the Mongolian cattle in the F group) and that the abundance of Firmicutes significantly decreased compared to that in the S group, which matched the results of the above study. The reasons for these changes are that in the grazing system, pastures contain large amounts of cellulose, hemicellulose, and structural carbohydrates which are difficult to ferment. Therefore, Mongolian cattle raised through grazing consume more plant fiber than those raised under housed feeding. These differences have led to changes in the rumen microbial composition, thus confirming the differences in the rumen flora under grazing and housed feeding conditions.

At the genus level, *unclassified__o___Bacteroidales, Prevotella*, *unclassified__c__Clostridia*, and *unclassified__f__Bacteroidacea* were the dominant genera in both groups in this study. Zhou et al. [32] reported that the relative abundance of *Prevotella* was significantly greater in the grazing and supplemental-feeding groups than in the forage-fed group. The relative abundance of *Ruminococcus* was lower in the F group than in the forage-fed and supplemental feeding groups. *Prevotella* is one of the most representative bacterial genera in the rumen biosystem, and it plays an important role in phytocellulose degradation [17]. Chiquette et al. [33] reported that supplementing dairy cows with *Prevotella* as a direct-fed microorganism significantly increased acetate concentration in the rumen. This suggests that the higher abundance of *Prevotella* in the rumen of Mongolian cattle in the F group may enhance cellulose degradation, leading to the production of greater amounts of acetate. This finding may indirectly explain the elevated acetate concentration observed in the F group. A study [34] found that *Ruminococcus*, a high-level energy group, plays a regulatory role in maintaining the stability of the rumen environment in yaks as the energy content of the ration increases. The results of this study revealed that in the F group, the abundance of *Prevotella* in Bacteroidota was increased and the abundance of *Ruminococcus* in Firmicutes was decreased. These findings suggested that Mongolian cattle are more capable of degrading cellulose from pastures under grazing conditions and that housed conditions help maintain the stability of the rumen environment, which could potentially have a positive impact on the health of Mongolian cattle.

The KEGG database is a comprehensive bioinformatics database that integrates genomic, chemical, and systemic functional information to reveal the genetic material and chemical blueprint of life forms. By annotating the KEGG functions related to rumen microbial function in Mongolian cattle with different feeding systems, we found that the KEGG function related to carbohydrate degradation was enriched in the rumens of the Mongolian cattle in the F group. Glycolytic pathways include starch and sucrose metabolism, galactose metabolism, and the interconversion of glucose and pyruvate. These findings suggested that Mongolian cattle in the F group are more capable of degrading carbohydrates and may produce more hydrolysis products and pyruvate. Mongolian cattle in the S group were enriched mainly in purine metabolism, pyrimidine metabolism, and peptidoglycan biosynthesis, as well as cell growth and death. Purines are abundant in organisms and are key raw materials for cell proliferation and important factors in immunomodulation [35]. Liu et al. [9] reported that feeding concentrates are more helpful for purine metabolism in the rumen of yaks. These findings suggest that purine metabolism is increased in the rumen of Mongolian cattle under housed feeding conditions, which may affect their growth and development.

CAZymes is a database resource on enzymes that can synthesize or degrade complex carbohydrates and sugar compounds. Ruminants can break down the cellulose structure of plant cell walls using carbohydrate-active enzymes secreted by microbiota in the rumen [36]. The synergistic action of multiple GHs on different parts of complex biopolymers facilitates the efficient degradation of plant fibers [23]. The results of this experiment revealed that the greatest relative abundance of GHs was associated with the F and S groups, accounting for 53.64% of all CAZymes, followed by GTs, CEs, CBMs, PLs, and AAs, reflecting the dominant ability and importance of the GH family in the degradation of fiber. Dai et al. [37] reported that cellulases belong to GH5, GH9, GH45, and GH48; two-thirds of hemicellulases belong to GH10, GH11, and GH26; and most of the oligosaccharide-degrading enzymes belong to GH1, GH2, GH3, and GH43, as determined by sub-transcriptomic analysis. In this study, Mongolian cattle in the F group presented greater cellulase (GH9), hemicellulase (GH10, GH28), and oligosaccharide-degrading enzyme (GH2) contents and greater xylan hydrolyzing activity enzyme (GH51) than Mongolian cattle in the S group. These findings suggested that, under grazing conditions, Mongolian cattle have a greater ability to degrade pasture, probably not only through enhanced cellulose degradation but also through enhanced hemicellulose degradation. The degradation of hemicellulose facilitates cellulose digestion in forage by exposing cellulose to microbial activity [38]. Previous studies [39] have indicated that a high abundance of *Prevotella* is typically associated with diets rich in plant-derived glycans and dietary fiber, and that *Prevotella* CAZy cluster enzymes, including GH51 and GH28, participate in the galactose degradation pathway. These findings suggest that the microbial community in Mongolian cattle from the F group exhibited an enhanced fiber degradation capacity due to increased expression and secretion of CAZymes involved in plant fiber breakdown. Specifically, the high abundance of the GHs family and the metabolic specialization of *Prevotella* allowed Mongolian cattle in the F group to efficiently utilize complex carbohydrates from pasture. This may partially explain the significant increase in galactose metabolism observed in the F group. The diets of Mongolian cattle under-housed conditions are rich in starch; therefore, the starch-degrading enzymes (GH13, GH31) are enriched in the S group. The rumen microbiota can better utilize starch and acquire more energy and other nutrients, which is beneficial to the growth and body maintenance of Mongolian cattle.

Metabolomics can reveal the role of microbial communities in ruminant digestion, absorption, and energy utilization and better understand host–microbe symbiosis. We evaluated the metabolites in the rumen fluid of Mongolian cattle in the F group and the S group via LC-MS metabolomic analysis and found that the samples from the F group and the S group were significantly separated, as determined by PLS-DA analysis and replacement tests, which confirmed that the different feeding systems significantly affected their rumen metabolism. We investigated the mechanisms underlying the effects of metabolites on host and rumen microbiota in Mongolian cattle under different feeding systems by integrating the rumen fluid metabolome and performing a correlation analysis of the dominant genera of rumen microbiota with differential metabolites. Subaphylline is an intermediate of arginine metabolism in arginine and proline metabolism (KEGG, map00330) and is formed by the combination of putrescine and ferulic acid through an enzyme-catalyzed reaction. Ferulic acid (FA) is a common phenolic acid in nongrain feeds such as pasture and straw, and Ferulic acid esterase (FAE) is an enzyme that catalyzes the ester-bond hydrolysis reaction between FA and acyl substrates, which efficiently releases FA from roughage substrates and promotes their crude fiber degradation ability in concert with other crude fiber-degrading enzymes (e.g., xylanase and cellulase) [40]. The upregulation of subaphylline in Mongolian cattle in the F group and the role of FAEs in crude fiber degradation in this study suggested that grazing increases the metabolites of digested cellulose in Mongolian cattle. Amino acids not only play important roles in the growth and metabolism of microbiota but are also the basic substances required for many important physiological processes, such as optimal growth, reproduction, normal lactation, and effective maintenance of body proteins in animals. Among organic acids and their derivatives, L-arogenate, an important intermediate metabolite in the biosynthesis of phenylalanine, tyrosine, and tryptophan, was downregulated in the rumens of Mongolian cattle in the F group (KEGG, map00400). Yi et al. [8] reported that a yak group with a higher ration concentrate ratio had relatively strong rumen amino acid metabolism. These findings suggested that compared to the F group, the S group presented greater rumen amino acid metabolism. To summarize, the metabolic changes were consistent with the metagenome results. The relationship between rumen microbial taxa and host metabolism was revealed by the joint examination of prevailing microbial genera and varying metabolites. *Prevotella*, Bacteroidota, and *Ruminococcus* were highly significantly associated with metabolites, among which included diglycerides related to lipid metabolism [DG (18:4(6Z,9Z,12Z,15Z)/15:0/0:0), and DG (13:0/20:3 (5Z,8Z,14Z)-O (11S, 12R)/0:0)] were significantly lower in the F group. A study [41] showed that diglycerides are important intermediates in fat synthesis. *Prevotella*, Bacteroidota, DG (18:4 (6Z,9Z,12Z,15Z)/15:0/0:0), and DG (13:0/20:3 (5Z,8Z,14Z)-O (11S,12R)/0:0) all showed highly significant negative correlations, whereas *Ruminococcus* was the opposite, suggesting that living conditions can promote fat synthesis in Mongolian cattle. The correlations shown by *Prevotella*, Bacteroidota, and *Ruminococcus* with these metabolites under different feeding regimes suggested that metabolite levels are influenced by microbiota abundance, thus affecting the regulatory functions of the organism. Among them, *Prevotella* and Bacteroidota were the key contributors to the rumen microbial metabolites of the Mongolian cattle in the F group, and *Ruminococcus* was the key contributor to the rumen microbial metabolites of the Mongolian cattle in the S group.

## 5. Conclusions

This study found that grazing and housed feeding significantly altered the rumen microbiota and metabolism of Mongolian cattle. Grazing enriched fiber-degrading bacteria (such as Bacteroidota and *Prevotella*) and GH2/GH10 enzymes, enhancing cellulose digestion, acetate production, and levels of fiber metabolites (such as Subaphylline), thereby optimizing the utilization of roughage. Housed feeding promoted the expression of starch-utilizing taxa (Firmicutes, *Ruminococcus*) and GH31 enzymes, activating purine and fatty acid metabolic pathways to facilitate starch decomposition and fat synthesis. These microbial-metabolic differences reflect the adaptive responses of Mongolian cattle to high-fiber and slightly more starch diets, providing a basis for improving feeding strategies and roughage utilization technologies.

## Figures and Tables

**Table 1:** Effects of different feeding systems on rumen fermentation parameters and cellulose-degrading enzyme activity in Mongolian cattle.

	Groups		SEM	*p*-Value
Items	F	S		
Ruminal pH	7.08	7.20	0.44	0.16
TVFA (mmol/L)	72.30	58.20	4.10	0.11
Acetate (mol/100 mol)	50.90 ^a^	38.13 ^b^	2.87	0.01
Propionate (mol/100 mol)	11.63	11.95	1.05	0.89
Butyrate (mol/100 mol)	6.03	6.52	0.59	0.71
Isobutyrate (mol/100 mol)	0.48	0.50	0.32	0.77
Valerate (mol/100 mol)	2.39	0.53	0.06	0.66
Isovalerate (mol/100 mol)	0.87	0.57	0.11	0.18
Cellulase (IU/L)	19.78 ^a^	18.08 ^b^	0.28	0.001
xylanase (U/L)	112.11 ^a^	101.59 ^b^	1.52	<0.001
β-glucosidase (U/L)	217.02 ^a^	195.67 ^b^	3.42	<0.001

Note: F, grazing; S, housed feeding; SEM, standard error of the means; TVFA, total volatile fatty acid. ^a,b^, in the same row, values with different superscripts, differ significantly (*p* < 0.05).

**Table 2:** Analysis of the α diversity of the rumen microbial community.

Items	Groups		SEM	*p*-Value
	F	S		
ACE index	1177.17	1055.50	32.781	0.06
Simpson index	0.14	0.13	0.005	0.67

Note: F, grazing; S, housed feeding; SEM, standard error of the means; ACE, abundance-based coverage estimator; Simpson, Simpson’s Diversity.

## Data Availability

The data of this experiment has been uploaded to the NCBI database, and the accession number is PRJNA1250165. https://www.ncbi.nlm.nih.gov/sra/PRJNA1250165 (accessed on 15 April 2025).

## Supplementary Materials

The following supporting information can be downloaded at: https://www.mdpi.com/article/10.3390/ani15121774/s1, Table S1: Nutritional levels of natural hay and composition and nutritional levels of the housed total mixed rations (dry matter basis); Table S2: Metagenomic sequencing results of Mongolian cattle with different feeding method. Table S3: Differentially metabolized substances in the rumen fluid of Mongolian cattle in the F group and the S group.

## Abbreviations

ACE	Abundance-based Coverage estimator
ADF	Acid detergent fiber
β-GC	β-glucosidase
CP	Crude protein
FA	Ferulic acid
FAE	Ferulic acid esterase
F	The grazing group
GAZymes	The carbohydrate active enzymes database
KEGG	Kyoto Encyclopedia of Genes and Genomes
M/Z	Mass-to charge ration
NDF	Neutral detergent fiber
ORF	Open read frame
P	Phosphorus
QC	Quality control
RSD	Relative standard deviation
RT	Retention time
S	The housed feeding group
SEM	Standard error of the mean
TMR	Total mixed ration
VIP	Variable importance projection
VFAs	Volatile fatty acids
