# Supplementary material for: Multi-Omics Revealed the Effects of Different Feeding Systems on Rumen Microorganisms, Cellulose Degradation, and Metabolites in Mongolian Cattle
Source: Animals (Basel). 2025 Jun 16;15(12):1774. doi: 10.3390/ani15121774 (PMC12189806; doi:10.3390/ani15121774)
Supplement: Supplementary file 1 [file animals-15-01774-s001.zip › animals-3674984-supplementary.pdf]

Table S1. Nutritional levels of natural hay and composition and nutritional levels of the housed total mixed rations (dry matter basis).

Items	Groups	
	F	S
Ingredients		
Silage corn		20.00
Corn stalks		40.00
Corn		26.00
Wheat bran		3.00
DDGS		5.00
Soybean meal		2.00
Rapeseed meal		2.00
$CaHPO_4$		0.50
$NaHCO_3$		0.50
Premix[1)]		1.00
Total		100.0
Nutrient levels%[2)]		
Crude protein	7.06	11.63
Neutral detergent fiber	65.94	44.58
Acid detergent fiber	40.44	28.85
Starch	6.40	24.36
Calcium	1.98	0.81
Phosphorus	0.15	0.38

F, grazing; S, housed feeding

[1)] The premix provides the following nutrients per kilogram of feed: VA 3 500 IU, VD 1 000 IU, VE 40 IU, Mn 40 mg, Fe 50 mg, Cu 10 mg, Zn 40 mg, and Se 0.30 mg.

[2)] Nutrient levels in the pastures and confine-fed diets were measured.

Table S2. Metagenomic sequencing results of Mongolian cattle with different feeding methods

Samples	Raw reads	Raw base(bp)	Clean reads	Clean base(bp)	Contigs	Contigs base(bp)	N50
F_1	102,046,864	15,409,076,464	100,639,794	15,159,976,225	1,678,305	1,013,702,457	609
F_2	86,761,290	13,100,954,790	85,105,820	12,822,245,902	1,485,201	865,026,689	587
F_3	86,536,062	13,066,945,362	84,924,648	12,798,653,399	1,384,817	823,400,150	595
F_4	88,273,046	13,329,229,946	85,972,376	12,956,065,995	1,522,540	855,485,140	556
F_5	90,554,498	13,673,729,198	88,123,428	13,282,695,823	1,481,577	834,346,217	559
F_6	81,462,326	12,300,811,226	80,301,760	12,107,039,851	1,320,867	754,872,238	572
S_1	87,248,380	13,174,505,380	85,504,286	12,853,131,451	1,380,622	899,805,983	659
S_2	84,400,856	12,744,529,256	81,800,788	12,331,584,922	1,303,476	859,895,422	678
S_3	87,975,590	13,284,314,090	86,739,302	13,078,024,745	1,427,478	952,401,147	688
S_4	87,462,012	13,206,763,812	85,312,202	12,861,359,776	1,359,261	906,142,267	688
S_5	86,528,558	13,065,812,258	85,545,068	12,875,137,763	1,257,714	880,137,786	731
S_6	81,462,326	12,352,786,634	80,430,368	12,114,240,179	1,350,036	874,345,683	661
Total	1,051,056,016	158,709,458,416	1,030,399,840	155,240,156,031	16,951,894	10,519,561,179	
Average	87,588,001	13,225,788,201	85,866,653	12,936,679,669	1,412,657	876,630,098	631

Note: Raw reads: number of raw sequence entries; Raw bases: total sequence length of raw sequences; Clean reads: number of sequence entries after QC; Clean bases: total sequence length after QC; Contigs: number of spliced sequence entries; Contigs bases: total sequence length of spliced sequences; N50: sequence lengths of contig sequences summed according to their lengths and sizes in ascending order. N50: the sequence length of each contig sequence in ascending order of length and the sequence length when the summed value exceeds 50% of the length of all sequences for the first time..

Table S3. Differentially metabolized substances in the rumen fluid of Mongolian cattle in the F group and the S group.

Super Class	Metabolite	P_value	VIP	FC	Trend
Lipids and lipid-like molecules	Verbenalol	<0.01	3.26	5.56	up
	Algestone	<0.01	3.16	3.21	up
	Zedoarondiol	<0.01	2.86	2.23	up
	4-Oxoretinol	<0.01	2.7	1.81	up
	Isocyperol	<0.01	2.68	1.78	up
	DG (18:4(6Z,9Z,12Z,15Z)/15:0/0:0)	<0.01	2.59	0.57	down
	4-Hydroxyretinoic acid	<0.01	2.55	1.77	up
	Gibberellin A8	<0.01	2.47	1.81	up
	Withaperuvin H	<0.01	2.41	1.57	up
	Desglucocoroloside	<0.01	2.31	1.61	up
	Alpha-Dimorphecolic acid	<0.01	2.3	0.64	down
	11b,13-Dihydrolactucin	<0.01	2.32	1.59	up
	Pregnanetriolone	<0.01	2.26	1.59	up
	(7b,10a)-3-Hydroxy-1,3,5-cadinatrien-9-one	<0.01	2.22	1.57	up
	DG (15:0/16:0/0:0)	<0.01	2.07	1.58	up
	Estrone	<0.01	1.99	0.61	down
Organoheterocyclic compounds	Daucol	<0.01	3.32	4.05	up
	Prodolic acid	<0.01	3.2	0.19	down
	Apitolisib	<0.01	2.92	2.12	up
	7-Methyl-2-(2-furyl)-1,8-naphthyridine-4(1H)-one	<0.01	2.76	0.6	down
	Enoxacin	<0.01	2.73	1.86	up
	Ketobemidone	<0.01	2.68	1.71	up

	(2R,3S,9R)-5-Acetyl-8,8-dimethyl-7,16-diazapentacyclo [9.6.1.02,9.03,7.015,18] octadeca1(17),11(18),12,14-tetraene-4,6-dione	<0.01	2.56	2.07	up
	1-Pyrrolidine carboxaldehyde, 3-(1,3-benzodioxol-5-ylmethylene)-2-oxo-	<0.01	2.43	0.61	down
	Cenisertib	<0.01	2.41	0.57	down
	LEUCOGENENOL	<0.01	2.27	1.63	up
	Sunepitron	<0.01	2.25	1.61	up
	4'-Hydroxymethohexital	<0.01	2.12	1.51	up
	5-Methylcytosine	<0.01	1.91	1.56	up
Organic acids and derivatives	N1-Acetylspermine	<0.01	3.17	0.32	down
	N-Acetyl-D-tryptophan	<0.01	3.08	0.33	down
	Hydroxypropyl-Tyrosine	<0.01	2.53	0.46	down
	4-Hydroxy-3-methoxy-cinnamoylglycine	<0.01	2.55	0.58	down
	L-arogenate	<0.01	2.54	0.66	down
	Glutaminyllysine	<0.01	2.45	1.93	up
	Cyclooctyl acetate	<0.01	2.41	0.66	down
	N-Malonyltryptophan	<0.01	2.34	0.59	down
	4-Amino-3-hydroxybutyrate	<0.01	2.28	1.51	up
	L-Histidine	0.03	1.62	1.65	up
Benzenoids	Chlorpropham	<0.01	2.8	0.45	down
	Salbutamol	<0.01	2.63	1.73	up
	Auramine	<0.01	2.6	0.53	down
	Phenylethylmalonamide	<0.01	2.32	0.6	down
	4-hydroxy-3-nitrophenyl acetate	<0.01	2.29	0.63	down
	2-(Malonylamino)benzoic acid	<0.01	2.29	0.66	down
	Bisoprolol	<0.01	2.26	1.57	up
	4-(2-Aminoethyl)-5-fluoro-1,2-benzenediol	<0.01	1.98	0.65	down

Phenylpropanoids and polyketides	3,4-Flavandione	<0.01	2.61	1.94	up
	Resveratrol	<0.01	2.18	1.56	up
	Subaphylline	<0.01	2.12	1.5	up
	13-Dihydrodaunorubicin	<0.01	2.07	2.11	up
	Medicarpin	<0.01	1.94	0.66	down
Alkaloids and derivatives	Senecionine N-oxide	<0.01	2.38	0.58	down
	Sparteine	<0.01	1.9	1.55	up
Nucleosides, nucleotides, and analogs	Abacavir	<0.01	2.93	0.27	down
	Cytochlor	<0.01	2.88	2.42	up
Organic oxygen compounds	Toxin FS2	<0.01	2.54	1.94	up
	N, N'-diacetyl chitobiose	0.04	1.59	1.52	up
Organic nitrogen compounds	Betaine aldehyde hydrate	<0.01	3.03	2.78	up
Organosulfur compounds	Dimethyl Sulfoxide	<0.01	2.4	1.83	up
Others	Cytisine	<0.01	2.44	1.55	up
	DG (13:0/20:3(5Z,8Z,14Z)-O (11S,12R)/0:0)	<0.01	2.37	0.66	down
	DG (20:4(5Z,8Z,11Z,14Z)-OH (18R)/i-13:0/0:0)	<0.01	2.35	0.6	down
	N-Arachidonoyl Arginine	<0.01	2.31	1.52	up

FC, Fold Change; VIP, The VIP value indicates the strength of the effect of between-group differences in the categorical discrimination of each group of samples in the model for the corresponding metabolite.
